# Editorial: Utilizing machine learning with phenotypic and genotypic data to enhance effective breeding in agricultural and horticultural crops

**DOI:** 10.3389/fpls.2026.1869724

**Published:** 2026-07-21

**Authors:** Jinyoung Y. Barnaby, Andrés J. Cortés

**Affiliations:** 1 U. S. Department of Agriculture, U. S. National Arboretum, Beltsville, MD, United States; 2 Facultad de Ciencias Agrarias—Departamento de Ciencias Forestales, Universidad Nacional de Colombia—Sede Medellín, Medellín, Colombia

**Keywords:** artificial intelligence (AI), big data, crop breeding, deep learning (DL), genotyping, germplasm, high-throughput phenotyping (HTP), pre-breeding

## Introduction

Environmental variability, agrobiodiversity loss, and market volatility are placing unprecedented pressures on agricultural systems (Hultgren et al., 2025), increasing the vulnerability of agricultural crops and horticultural trees, and shifting market preferences (Benitez-Alfonso et al., 2023). Rising temperatures have contributed to prolonged droughts (López-Hernández et al., 2023) and heat (López-Hernández et al., 2025c) waves, along with shifts in precipitation patterns, increased soil salinity (Guo et al., 2023), heightened pest and pathogen pressures (Guevara-Escudero et al., 2021), all of which severely threaten crop productivity and stability. Shifts in seasonal patterns and extreme temperatures affect not only yield but also food quality (Abberton et al., 2016). In turn, consumer narrow preferences, and market volatility and erratic prices of major staple and commodity crops, add uncertainty to an already highly vulnerable scenario (Smale and Jamora, 2020). In response, plant breeding efforts are focusing on developing environmentally resilient varieties capable of withstanding these multifaceted challenges (Campbell et al., 2025).

In this context, the development of varieties resilient to varying environmental conditions, along with market-versatile crop types (Peláez et al., 2022), is recognized as essential to mitigate these challenges while ensuring global sustainable food security (Benitez-Alfonso et al., 2023). Historically, breeding for complex traits, such as abiotic and biotic stress tolerance, yield and quality, and water- and nutrient-use efficiency, has relied on phenotypic recurrent selection, which more recently has been complemented by marker-assisted selection (MAS) (Pinson et al., 2022; Bedoya-Londoño et al., 2025) derived from simple sequence repeat (SSR) markers (Warnke et al., 2023), and quantitative trait loci (QTL) mapping (Ahmar et al., 2021; Fernandez-Baca et al., 2021a; Barnaby et al., 2022; Cortés, 2024). With the advancement in computing capabilities and high-throughput genotyping, particularly next-generation sequencing (NGS), genome-powered solutions have come into play by enabling a more precise identification of quantitative trait nucleotides (QTNs) associated with complex traits (Vega-Muñoz et al., 2025), as well as their prediction through genomic selection (GS) across environments and complex genetic backgrounds (Arenas et al., 2025).

Despite substantial advances in sequencing technologies, analytical pipelines, and computer power, the systematic phenotypic evaluation of large populations under field conditions remains a major limitation (Mir et al., 2019). Traditional phenotyping methods are laborious, time- and budget-consuming, and often subjected to observer bias and environmental inconsistencies. Furthermore, phenotype and adaptive potential forecasting remain a major bottleneck due to its poor precision, variability, and complex interactions (Li et al., 2025). ML and AI now offer transformative potential in overcoming these challenges from the plant genetics perspective (Ma et al., 2014b) as well as the crop breeding discipline itself (Varshney, 2021). ML and AI can capture complex, nonlinear relationships between genotypes, phenotypes, and environmental factors (Libbrecht and Noble, 2015; Schrider and Kern, 2018), leveraging in this way computational power to analyze large datasets (Tong and Nikoloski, 2021). This integration not only promises to accelerate the breeding pipeline but also is likely to enhance the precision of trait selection, particularly for complex—often plastic—polygenic traits (Arenas and Cortés, 2026), including the adaptive potential (Cortés, 2025), and traits influenced by soil microbial associations (Fernandez-Baca et al., 2021b, 2021).

**Figure 1:**
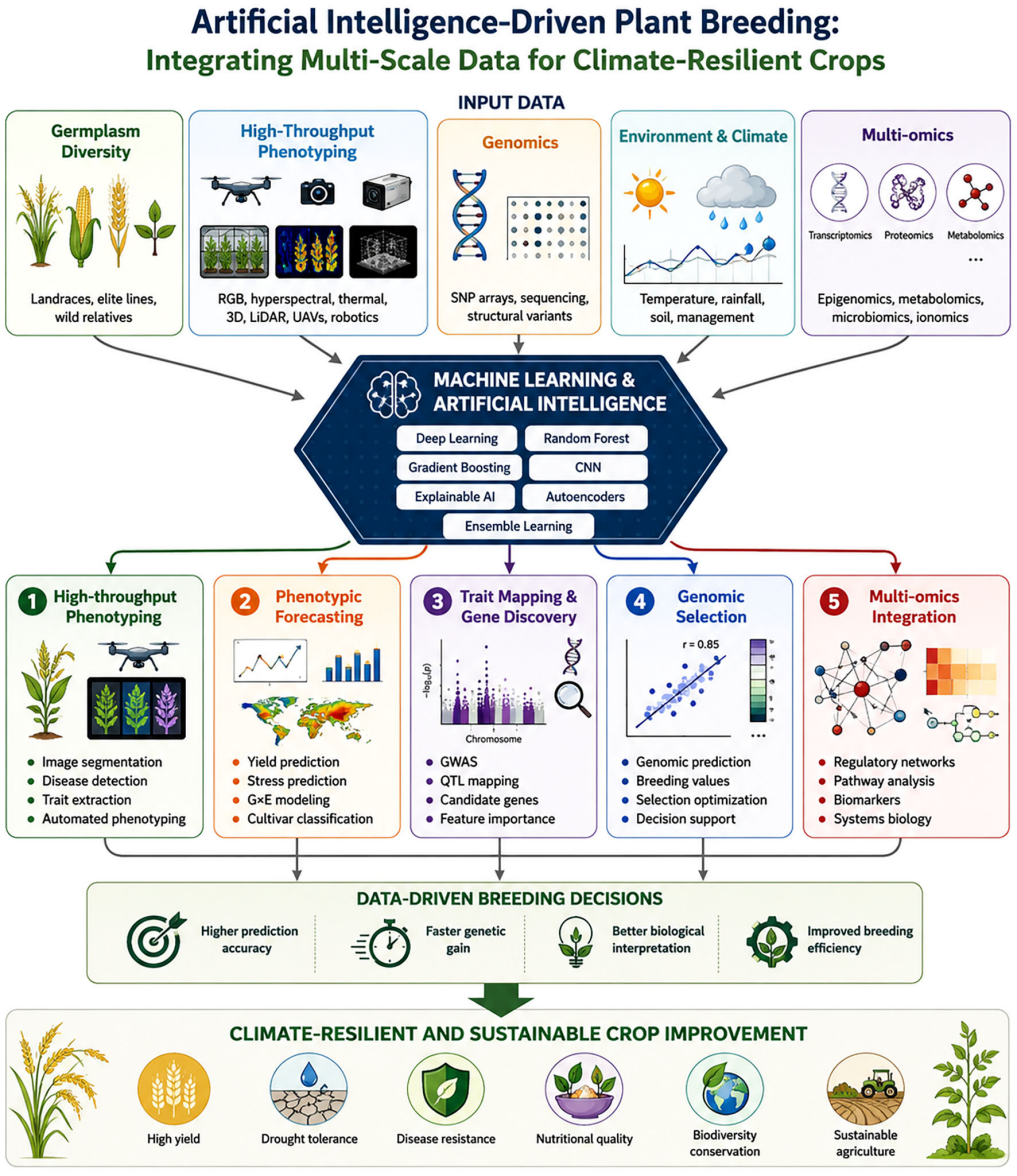
Conceptual, AI-inspired summary of the applications of machine learning (ML) in modern plant breeding, illustrating their roles across five research domains: high-throughput phenotyping, phenotypic forecasting, trait mapping and candidate gene identification, genomic selection, and multi-omics integration. The figure synthesizes key ML applications featured in the Research Topic, “Utilizing Machine Learning with Phenotypic and Genotypic Data to enhance Effective Breeding in Agricultural and Horticultural Crops”, highlighting how AI-driven approaches enhance prediction accuracy, accelerate trait discovery, and support data-informed breeding decisions (see **Table 1** for more details).

Given the rapid advances in molecular crop breeding, this Research Topic on “Utilizing Machine Learning with Phenotypic and Genotypic Data to enhance Effective Breeding in Agricultural and Horticultural Crops” highlights how ML and AI can scale phenotyping, genotyping and their integration across modern breeding applications. Specifically, the Research Topic aims to compile innovative case studies where advanced phenotyping and genotypic tools are integrated through ML, AI, and deep learning (DL) to enhance breeding efficiency. The 15-contributing works (**Table 1**), spanning a diverse array of study systems, illustrate how these analytical technologies can enhance phenotyping, trait prediction, gene discovery, genomic selection, and multi-omics integration (**Figure 1**). These contributions collectively illustrate how ML functions not only as data-analysis tool but also catalyst of modern crop improvement. Specifically, ML/AI help overcome persistent bottlenecks in scalable, objective phenotyping and in forecasting complex genotype–environment interactions, complementing GS and GWAS pipelines and supporting the shift toward data-integrated breeding.

**Table 1:** Summary of the 15 studies compiled within this Research Topic, “Utilizing Machine Learning with Phenotypic and Genotypic Data to enhance Effective Breeding in Agricultural and Horticultural Crops”.

Species	Key trait(s)	Main goal	ML/AI method(s)	Key result(s)	Perspective(s)	Ref.
ML-assisted high-throughput phenotyping and imaging technologies						
Buckwheat (*Fagopyrum esculentum*)	Seed traits (shape, size, color)	Study inheritance of seed traits via image-based phenotyping	RGB imaging + ImageJ + Statistical analysis	Maternal inheritance of shape/color, overdominance in size	Integrating AI-based segmentation and multimodal imaging	Oh et al.
Soybean	Yield	Predict soybean yield from RGB images	GBDT best among 5 ML algorithms	R^2^ = 0.82 with multi-angle, multi-type features	ML-driven phenotyping for scalable yield prediction	Li et al.
Barley	Pathogen interaction	High-throughput microscopy for pathogen interaction	BluVision Micro with RF classifiers & CNNs	30× faster than HyphArea, GWAS linked to resistance loci	Accelerate disease resistance screening with ML tools	Lück et al.
Bentgrass (*Agrostis stolonifera* × *A. capillaris*)	Tiller number	Automated instance-level tiller quantification in hybrid populations	YOLOv8 (one-stage detector) *vs*. Faster R-CNN & edge-based segmentation	YOLOv8 achieved R² = 0.97, highest accuracy and fastest inference, robust to dense occlusion	YOLOv8 enables scalable instance-level phenotyping for downstream genetic analysis & breeding	Ferm et al.
Machine learning models for phenotypic forecasting						
Lychee	Fruit traits & cultivar ID	Phenotyping and cultivar classification	CNN-based segmentation models + SVM, RF, LDA	Segmentation Dice >0.90, LDA accuracy ~79%	Automated fruit analysis and cultivar ID with multimodal imaging	Xue et al.; Kim et al., (2024)
Maize	Days to pollen, yield	Trait prediction across environments	Compositional Autoencoder (CAE)	7×-10× better accuracy than PCA/PLSR	Latent feature disentanglement Enables precise trait prediction	Powadi et al.
AI and ML in trait mapping and candidate gene identification						
Sorghum	Callus induction, regeneration	Identify QTLs for *in vitro* culture traits	Multi-locus GWAS via ML-based methods	34 QTLs + 47 candidate genes identified	ML-GWAS for complex traits and *in vitro* breeding	Xu et al.
Wheat	Plant height	GWAS for plant height in wheat	MLM with admixture & kinship correction	44 SNPs identified, 7 linked to key genes	ML-GWAS to dissect polygenic traits in pre-breeding	Ding et al.
ML-enabled genomic selection						
Rice, sunflower, wheat, maize	Multiple agronomic traits	Enhance genomic selection with deep learning	Parallel CNN with multi-kernel design	+0.031 accuracy over DNNGP in 24 traits	Future work on data balancing and multi-trait GS	Xie et al.
Almond	Shelling fraction	Predict shelling fraction with XAI	RF + SHAP for SNP interpretation	RF R² = 0.511, key SNP linked to seed development	ML-XAI integration for better GS interpretability	Novielli et al.
Coffee	Yield, fruit number, pest and disease resistance	Boost GS with ensemble learning	SEL (GBLUP, RF, MARS, QRF + Meta-learners)	Up to 200% gain in trait prediction	SEL captures complex trait architecture in GS	Nascimento et al.
Multi-omics integration and predictive breeding						
Soybean	Protein content	Identify bHLH genes linked to protein synthesis	Comparative genomics + expression profiling	Several bHLH genes linked to protein regulation	Gene editing for seed quality improvement	
Wheat	Flower development	Study MADS-box gene evolution and function	ML-based clustering + expression profiling	Identified 15 TaE genes, functional validation	Targeted editing for flower traits and yield	Bai et al.
*Brassica napus*	Flower color (anthocyanin content)	Decipher anthocyanin-based petal color regulation	WGCNA + transcriptomics + metabolomics	MYB75-F3H module linked to color variation	WGCNA enhances trait discovery in breeding	Cui et al.
*Impatiens uliginosa*	Flower color	Explore factors driving flower color	Multi-omics + physiological assays	Anthocyanins + CHS expression drive color	Candidate targets for breeding via MAS/gene editing	Zhao et al.

The studies highlight how machine learning (ML) and artificial intelligence (AI) are being applied across high-throughput phenotyping, phenotypic forecasting, trait mapping, genomic selection, and multi-omics integration to support modern plant-breeding efforts.

## Advances in ML-assisted high-throughput phenotyping

High-throughput phenotyping (HTP) systems, powered by advancements in sensor technologies and imaging analysis, have revolutionized the way phenotypic data is collected and analyzed (Juliana et al., 2019; Mir et al., 2019; Barnaby et al., 2020; Kim et al., 2024). Imaging platforms, including RGB cameras, multispectral and hyperspectral imaging, thermal cameras, and 3D laser scanners, all allow for the non-invasive assessment of plant traits at various growth stages (Volpato et al., 2021; Gimode et al., 2023; Kim et al., 2024). Machine learning algorithms, particularly convolutional neural networks (CNNs), have shown remarkable success in analyzing complex image data for phenotypic traits (Xue et al.). These methods have been used for disease detection, biomass estimation, canopy structure analysis, and stress symptom identification in various crops (Cortés and Barnaby, 2023; Li et al.; Oh et al.). For instance, CNN-based models have achieved high accuracy in detecting foliar diseases in barley (Lück et al., 2025), predicting cultivar phenotypes in lychee (Xue et al.), and assessing agronomic traits in rice, sunflower, wheat and Maize (Xie et al.). In turn, deep learning models have enabled the extraction of subtle features from images that may not be discernible through traditional image processing techniques. This capability further enhances the reliability and repeatability of phenotypic measurements, which is critical for large-scale breeding programs.

To exemplify the power of ML for image processing, the study by Oh et al. in this Research Topic used high-throughput image-based ML phenotyping to explore the genetic inheritance of seed traits (*i.e.*, shape, size, and coat color) in common buckwheat (*Fagopyrum esculentum*). Researchers captured RGB images of over 100 F_1_ seeds from diverse germplasm, and systematically extracted quantitative features (*i.e.*, area, length, width, aspect ratio, and grayscale) using *ImageJ* software, enabling precise and scalable trait measurement. Statistical analysis revealed that seed coat color and shape exhibit maternal inheritance, while size parameters often showed overdominance, especially in crosses with contrasting parental seed sizes. The study also highlighted potential barriers for intercrossing linked to flower type and parent combinations. Through this work, the authors suggest integrating ML and advanced imaging techniques, such as AI-based segmentation and multimodal data analysis, to enhance phenotyping efficiency and accuracy, supporting breeding programs aiming for superior buckwheat varieties.

Similarly, the study by Li et al. also explored high-throughput RGB with ML to non-destructively estimate individual soybean yield at the R6 stage. Authors successfully extracted morphological, color, and textural features from over 18,000 top- and side-view images of 240 diverse soybean genotypes. To accomplish so, they applied feature selection at Pearson correlation ≥0.5 and tested five ML algorithms (*i.e.*, CatBoost, LightGBM, Random Forest, GBDT, and MLP). The team found that Gradient Boosted Decision Trees (GBDT) was the best performer, achieving an *R²* of 0.82, especially when combining multi-angle, multi-type features. Therefore, this highly scalable ML-driven phenotyping approach offers an accurate prediction of yield in soybean. Authors predict further improvements in the prediction ability of GBDT by adding sensor types, expanding image acquisition across growth stages, and refining feature selection.

Machine learning is also capable of assisting phenotyping at microscopic scales. Specifically, writing for this Research Topic, Lück et al. presented BluVision Micro, a modular, open-source phenotyping platform that leverages machine learning and automated microscopy for detailed, high-throughput analysis of microscopic plant-pathogen interactions. The platform integrates both custom-made feature models (*e.g.*, Random Forest classifiers) and convolutional neural networks (CNNs) to detect and quantify fungal microcolonies on barley leaves with high speed, up to 30× faster than the predecessor HyphArea. The tool also provides interpretable outputs via CNN heatmaps. Authors tested the pipeline on 196 diverse barley genotypes inoculated with powdery mildew. The software recorded statistics like colony count and area that were integrated into a GWAS model, which identified several associations enriched in defense-related genes. This tool offers a scalable, accurate disease-resistance screening, ultimately unveiling novel resistance loci.

To further highlight the versatility of ML-enabled phenotyping, the study by Ferm et al. in this Research Topic developed a high-throughput, deep learning–based workflow to quantify tiller number in interspecific bentgrass hybrids—a trait traditionally difficult to measure because of the thin, filamentous morphology of tillers and their frequent occlusion. Using controlled imaging of clipped tillers placed on standardized high-contrast backgrounds, the authors compared a classical edge-based segmentation pipeline with two convolutional neural network object detectors, Faster R-CNN and YOLOv8. Although the two-stage Faster R-CNN model showed higher localization precision, the one-stage YOLOv8 detector provided markedly superior total-count accuracy, achieving an overall R² of 0.97 while maintaining robust performance even in densely crowded samples exceeding 400 tillers per image. The study also demonstrated YOLOv8’s computational efficiency, completing training in ~4 hours compared with ~26 hours for Faster R-CNN, and revealed that recall-oriented one-stage detection is better suited for fine, overlapping plant structures where minimizing missed instances is more critical than boundary tightness. Altogether, this workflow provides the first scalable, instance-level solution for bentgrass tiller quantification and establishes a transferable phenotyping framework capable of generating high-quality trait data for downstream genetic and breeding applications. Complementary to this study, a low-cost automated greenhouse imaging platform integrating ML-based processing was developed to evaluate drought tolerance in bentgrass hybrids (Kim et al., 2024), demonstrating how accessible, controlled-environment phenotyping systems can generate high-frequency, reproducible trait data.

## Machine learning models for phenotypic forecasting

Machine learning encompasses algorithms for regression, classification, clustering, and prediction, making it adaptable to many plant breeding applications (Tong and Nikoloski, 2021). Among these, (*i*) regression algorithms speed up the detection of proxy traits and age-age correlations with higher precision (Li et al.), (*ii*) unsupervised classification and clustering algorithms enable forecasting trait segregation in germplasm collections with genepool and racial substructure (Schrider and Kern, 2018; Park et al., 2020; Xue et al.; Denning-James et al., 2025; López-Hernández et al., 2025a), and (*iii*) supervised training of target breeding labels with environmental features simplify multi-locality field trials (Azodi et al., 2019; Powadi et al.) by directing them toward specific gaps within the phenotypic-environment continuum (Costa-Neto et al., 2020; Costa-Neto and Fritsche-Neto, 2021; Resende et al., 2021).

Commonly used algorithms include decision trees, random forests, support vector machines (SVM), gradient boosting machines (GBM), and deep neural networks (DNN) (Libbrecht and Noble, 2015). More recently, ensemble learning methods, which combine multiple algorithms to improve predictive performance, have gained popularity in genomic prediction studies (Abdollahi-Arpanahi et al., 2020; Banerjee et al., 2020). Ensemble techniques such as stacking and bagging enhance model robustness by reducing variance and bias (Nascimento et al.). In breeding programs, these methods have been applied to predict complex traits like yield (Li et al.; Nascimento et al.), drought tolerance, and disease resistance (Lück et al.) with improved accuracy over traditional statistical models. At the frontier of the field, explainable AI (XAI) approaches are increasingly being integrated into ML models to enhance their transparency and interpretability (Novielli et al.). XAI tools help breeders understand which features, such as phenotypic measurements, environmental variables, or specific genetic marker, are driving model fitting. This insight is invaluable for refining feature selection criteria and making informed breeding decisions.

Concerning trait clustering approaches, the study by Xue et al. developed an advanced deep learning pipeline for non-destructive phenotyping and cultivar classification in lychee using photon-counting micro−CT imaging. Researchers built and compared seven CNN-based segmentation models, including UNet variants (UNet/UNet++/UNet3+), AttentionUNet, SegNet, TransUNet, and DeepLabV3+, to automatically segment internal fruit structures (*i.e.*, kernel, pulp, epicarp, endocarp) with exceptional accuracy (Precision ≈ 0.90–0.99). Authors further extracted morphological features to distinguish lychee species via classifiers like SVM, Random Forest, and LDA. The LDA model, using leave-one-out cross-validation, achieved ~78–79% classification accuracy, demonstrating the efficacy of combining deep learning-based feature extraction and classical ML for both phenotyping and cultivar discrimination. This approach enables automated fruit trait analysis and cultivar identification, with further improvements expected from transfer learning and multimodal imaging.

Regarding ML-assisted trait forecasting across environments, Powadi et al. introduce a cutting-edge compositional autoencoder (CAE) to disentangle genotype- and environment-specific latent features from high-dimensional maize hyperspectral data, representing a significant advancement in ML-driven plant trait prediction. Autoencoders are a type of artificial neural networks used to learn latent features (*i.e.*, compressed representations of data) by training the network to reconstruct its own input. This being said, authors structured the CAE to partition latent representations into genotype, macro−environment, and micro−environment components. They tested the workflow on data from 578 maize inbreeds over multiple field environments and demonstrated that CAE enhances predictive accuracy by approximately sevenfold for “Days to Pollen” and tenfold for “Yield” compared to traditional principal components analyzes (PCA) and partial-least square regression (PLSR) autoencoders. This ML pipeline enables precise trait prediction across contrasting environments and opens new avenues for integrating sensor data, multimodal inputs, and time-series measurements to accelerate breeding (Sadohara et al., 2021).

## Application of AI and ML in trait mapping and candidate gene identification

Recent studies have demonstrated the power of AI and ML in trait mapping and candidate gene identification (Payseur et al., 2016). For example, ML-based multi-locus genome-wide association studies (GWAS) could successfully identify significant QTLs related to callus induction and regeneration in crops. In particular, writing for this Research Topic, Xu et al. examined 236 sorghum mini−core lines using mature seeds to quantify four key *in vitro* tissue culture traits (*i.e.*, callus induction, embryogenic callus formation, browning, and plant regeneration). Authors conducted multi−locus GWAS analyzes with over 6 million SNP markers, by relying on machine learning-based GWAS methods that, unlike traditional single-locus GWAS, are designed to capture complex genetic architectures by considering multiple loci simultaneously. These models, implemented in the mrMLM (*i.e.*, multi-locus random-SNP-effect mixed linear model) package in R, include multiple multi-locus approaches such as mrMLM, FASTmrMLM, FASTmrEMMA, pLARmEB, pKWmEB, and ISIS EM-BLASSO. They identified five genotypes (*i.e.*, IS5667, IS24503, IS8348, IS4698, and IS5295) exhibiting high regeneration potential, mapped a total of 34 QTLs (eight for callus induction, one each for browning and embryogenesis, and 24 for differentiation), and pinpointed 47 candidate genes within these regions. Fourteen of these genes have known orthologs implicated in cellular reprogramming and embryogenesis (including WIND1, which enhances callus and shoot formation). These integrative approaches also identified candidate genes, including transcription factors and kinases involved in cellular maintenance and embryogenesis. Such findings improve the prospects for *in vitro* cultivation, and downstream applications like gene editing (Doudna and Charpentier, 2014).

Also at the frontier of ML-assisted GWAS mapping, Ding et al. used an ML-based approach through mixed linear models (MLM) with admixture (*Q*) and kinship (*K*) correction as proxies to control for confounding factors like population stratification and relatedness. Authors analyzed over 55,000 SNP markers across 239 global wheat varieties grown in four environments. The GWAS models identified 44 significant SNPs on 12 chromosomes associated with plant height, with some explaining up to 11% of phenotypic variance. Crucially, seven loci were linked to candidate genes involved in photosynthesis, ion transport, transcription regulation, and protein degradation pathways. This work offers insights into the polygenic nature of wheat plant height and highlights the power of machine-learning driven GWAS for marker discovery and wheat pre-breeding. These findings reinforce the potential of ML in unraveling the genetic basis of complex traits, and accelerating the development of improved environmentally resilient and sustainable varieties (Grinberg et al., 2020).

## Integration of phenotypic and genotypic data for ML-enabled genomic selection

The integration of high-throughput phenotypic data with genotypic information represents a significant advancement in modern breeding strategies, especially when selecting polygenic environmentally shaped complex traits (Qiu et al., 2016). Genomic selection (GS), which predicts the breeding value of individuals based on genome-wide markers (López-Hernández et al., 2025a), has been enhanced through the incorporation of phenomic data and more robust genotyping platforms (Tong and Nikoloski, 2021).

A milestone in the rapidly evolving field of ML-assisted GS is the work by Xie et al., who introduced PNNGS, a novel parallel convolutional neural network designed to substantially improve genomic selection accuracy. Unlike serial deep-learning models, PNNGS processes high-density SNP data through multiple convolutional branches that operate in parallel using different kernel sizes (*e.g.*, 1×1, 3×3, 5×5, 7×7). PNNGS was tested against RR-BLUP, random forests, SVR, and a serial deep neural network (DNNGP) on 24 phenotypic labels across rice, sunflower, wheat, and maize. PNNGS consistently outperformed the other models, showing an average increase of 0.031 in prediction accuracy over DNNGP. Major advantages of PNNGS include parallel deep-learning architectures, use of residual learning, custom loss functions, and cluster-aware sampling. Still, authors acknowledge that future work should expand data-balancing strategies and test PNNGS across additional species and traits.

This Research Topic also demonstrates how advanced ML models and explainable AI (XAI) techniques improve the interpretability of genomic-enabled trait prediction. Specifically, the study by Novielli et al. tested this approach using a key agronomic fruit trait referred in almond as the “shelling fraction”—*i.e.*, the ratio of edible kernel to whole fruit. They began by using high-dimensional SNP data from 98 almond cultivars, applying feature selection and training multiple ML regression models (*i.e.*, Random Forest, Gradient Boosting, and AdaBoost, together with gBLUP and rrBLUP) to predict the shelling fraction. The Random Forest model outperformed others with an *R^2^* of 0.511 ± 0.025. Authors applied XAI via SHAP (SHapley Additive exPlanations) to better interpret model outputs and identify the most significant SNP markers. Interestingly, the top-ranked SNP mapped to a gene implicated in seed development. The work illustrates how integrating ML prediction with XAI enhances the accuracy of genotype-phenotype predictive models while offering a mechanistic understating of the underlying nature, boosting GS utilization in crop breeding.

Not being these major accomplishments in ML-assisted GS exciting enough, Nascimento et al. further released in this Research Topic an innovative stacking ensemble learning (SEL) framework designed to enhance GS by integrating through a meta-learner (*a.k.a.*, blender) predictions from multiple models (*i.e.*, base learners). Specifically, authors used the coffee tree (*Coffea arabica*) as proof of concept to demonstrate substantial improvements in the combined prediction of key traits, including yield, fruit number, leaf miner infestation, and cercosporiosis incidence. They used 5,970–21,211 SNPs from 195 coffee individuals to train GBLUP, MARS, Quantile Random Forests, and Random Forest, and merge their predictions via meta-learners such as Ridge Regression and two-kernel GBLUP within the SEL architecture. The SEL models outperformed all individual models, showing predictive ability gains of 87% for yield, 38% for fruit number, 200% for leaf miner infestation, and 15% for disease incidence compared to standard GBLUP. This approach captures complex genetic and omnigenic architectures (Boyle et al., 2017) by integrating multiple ML methods within a layered ensemble, rather than relying on comparisons among single models.

Multi-trait genomic prediction models could also leverage phenotypic data collected through HTP platforms to improve the accuracy of genomic-estimated breeding values (Guo et al., 2020; Lenz et al., 2020). So far, ML algorithms have facilitated the integration of these diverse datasets by handling high-dimensional data and identifying complex interactions between genetic markers and phenotypic traits. This approach allows for the discovery of novel marker-trait associations capable to handle second-order interactions like pleiotropic and epistatic effects (Haleem et al., 2022). More recently, integrating spectral reflectance indices with genomic data has additionally improved the prediction of yield and protein content under varying environmental conditions (Wu et al., 2025).

## Multi-omics integration and predictive breeding in the era of ML

Beyond genomics and phenomics, integrating multi-omics datasets (Cortés and Du, 2023), like transcriptomics (López-Hernández and Cortés, 2022; 2026), proteomics (Castillejo et al., 2023), metabolomics (Henao-Rojas et al., 2022; Tienda-Parrilla et al., 2022; Cui et al.), epigenomics (Mehrmohamadi et al., 2021; Du et al., 2025), and more recently metagenomics (Zhang et al., 2021; Nwachukwu and Babalola, 2022), pangenomics (Hu et al., 2025) and enviromics (Cooper and Messina, 2021; Costa-Neto et al., 2021; Resende et al., 2021; Bedoya-Canas et al., 2024; López-Hernández et al., 2025b), provides a more complete understanding of complex trait architecture (Cortés et al., 2023). Machine learning models (Ma et al., 2014b; Myburg et al., 2019), particularly DL architectures, are well suited to integrating these complex, high-dimensional, and heterogenous datasets (Barrera-Redondo et al., 2020).

At the interface of genomics and transcriptomics, the contribution to this Research Topic by Wu et al. reports a comprehensive genome−wide identification and expression profiling of the basic helix–loop–helix (bHLH) transcription factor family in soybean, elucidating their potential role in regulating grain protein synthesis. A total of 188 Gm bHLH genes were identified, and detailed analyzes of their phylogenetic relationships, gene structures, conserved motifs, chromosomal localizations, and promoter cis−elements were done. Expression profiling during seed development revealed several Gm bHLH genes that were differentially regulated and showed strong correlations with specific stages of protein accumulation. Notably, a subset of these genes showed strong associations with known pathways involved in nitrogen metabolism and storage protein regulation. The study highlights promising candidate bHLH genes for future functional validation, offering a candidate for genetic manipulation of protein content and nutritional quality in soybean seeds.

Following a similar analytical roadmap, the study of Bai et al. in this Research Topic reports a genome-wide identification and characterization of the E-class MADS-box gene family in wheat my integrating comparative genomics, expression profiling, and interaction assays to unravel their evolutionary trajectory and roles in flower development. Authors identified 15 TaE genes in bread wheat and 9 orthologs in ancestral relatives, classifying them into three phylogenetic subgroups and demonstrating conserved structural motifs and nuclear localization. The study leveraged ML-based clustering for phylogenetic tree construction and collinearity/synteny analysis to trace how polyploidization and gene duplication shaped this family. Expression data highlighted spike- and floret-specific activity, notably of TaSEP5-A, which functional role and interactions with other TaEs were respectively verified through *Arabidopsis* transformation and yeast two-hybrid assays. Being flower development a key fitness trait, authors envision utilizing gene editing to manipulate flowering time and structure to enhance wheat yield and adaptability across varying environmental conditions.

At the exciting interface of transcriptomic and metabolomic multi-omic data integration, the study by Cui et al. investigates the molecular basis of flower color variation in *Brassica napus* spanning an impressive total of eight petal colors. Authors captured 275 anthocyanin-related genes via BLAST and performed RNA−seq, revealing significant upregulation of structural genes like F3H and UGT, and transcription factors including MYB75, GL3, and TTG1. The team further applied a weighted gene co-expression network analysis (WGCNA), which is a ML-based network clustering algorithm. This technique enabled authors to pinpoint core omnigenic (Boyle et al., 2017) regulators (*i.e.*, CHS, F3H, MYB75/12/111) associated with anthocyanin accumulation in colored petals. This combined ML-driven transcriptome-metabolome approach suggests that MYB75-mediated activation of F3H plays a central role in pigment accumulation. This work not only deciphers transcriptional regulation underlying flower color variation, but also offers candidate targets for breeding ornamental varieties. ML tools such as WGCNA can accelerate trait discovery by identifying patterns across large omic datasets. Integrating transcriptomic and metabolomic data with phenotypic information could further improve the prediction of environmentally responsive traits in breeding programs. For instance, multi-omics integration has been applied successfully in legumes, perennial crops, and cereals to identify biomarkers for stress tolerance and quality traits (Villordo-Pineda et al., 2015; Espichan et al., 2022; Loupit et al., 2022).

Zhao et al. further explored multi-omic regulation of flower color, examining how cellular, physiological, biochemical, and genetic factors underly the distinct pigmentation patterns (*i.e.*, deep red, red, pink, and white) in *Impatiens uliginosa*. The team combined a comprehensive set of methodologies, including colorimetric analyzes, microscopy of epidermal cells, vacuolar pH measurement, pigment quantification (*i.e.*, anthocyanins and flavonoids), chemical profiling of key pigments (*i.e.*, malvidin−3−galactoside, cyanidin−3−O−glucoside, delphinidin), and gene expression assays of chalcone synthase (IuCHS) via qRT−PCR. Authors found that (*i*) anthocyanins dominate pigment composition with highest concentrations in red and pink flowers, (*ii*) cellular pH values correlate with color intensity, and (*iii*) IuCHS expression peaks in pink flowers, reaching its lowest level in white ones. Biochemical differences, especially in anthocyanin levels and CHS gene activity, are central to color variation, even though epidermal cell structures are similar among the different flower types. Overall, this multi-omics approach clarifies the physiological and molecular mechanisms that can guide breeding of new *Impatiens* varieties through MAS and gene editing targeting CHS and anthocyanin pathways.

Despite these promising research avenues in the integration and understanding of regulator networks across multiple omics layers (Du et al., 2025), challenges remain in harmonizing data collected from different omics platforms and ensuring the scalability of models across diverse populations and environments (Bedoya-Canas et al., 2024; Juma et al., 2025). Nevertheless, advances in systems biology and computational data science continue to drive rapid progress in this field (Myburg et al., 2019).

## Perspectives

Despite the advances highlighted in this Research Topic, several challenges must be addressed to release the full potential of AI/ML in crop breeding. First, data quality and standardization are often overlooked (Waldvogel et al., 2020), yet are essential for ensuring consist and accurate phenotypic and genotypic datasets, which are crucial for model training, testing and validation. Second, ML models must be validated across multiple environments and populations within a breeding program (Cortés et al., 2024) to ensure robustness and generalizability (Zhang et al., 2019; Thistlethwaite et al., 2020). Third, despite major increases in computational capacity over recent decades, integrating large-scale multi-omic datasets requires even greater computational infrastructure and more efficient data-compression methods to enable scalable data storage and processing (Barnaby et al., 2013; Barnaby et al., 2015; Kim et al., 2016; Barnaby et al., 2019a; Barnaby et al., 2019b; Wang et al., 2022; Barnaby et al., 2025). In addition, the rapid evolution of the field raises ethical considerations that have not yet fully addressed, including questions of data and model ownership, and equitable access to AI-driven breeding technologies, both of which are critical for fair global agricultural development (Cortés, 2025).

Addressing these challenges will require progress not only in technical fronts (Ma et al., 2014a; Qiu et al., 2016; Sadohara et al., 2021; Sousa et al., 2021) but also in societal and policy dimensions (Louwaars, 2019; Peláez et al., 2022; Kholova et al., 2024). Specifically, more scalable and cost-effective phenotyping platforms will help match the pace at which genomics has progressed (Khaipho-Burch et al., 2023). Incorporating real-time environmental data and sensor networks will further enhance the predictive capacity of breeding models. Similarly, refining ML algorithms for greater interpretability—not just usability—will help shift the emphasis from predictive performance alone to also understanding the underlying mechanisms. Finally, promoting fair and collaborative data-sharing frameworks (*e.g.*, Mccouch et al., 2016; Spindel and Mccouch, 2016) will help balance advances in computational capabilities with on-farm phenotyping needs and market realities in the global South (Santantonio et al., 2020), which is essential for developing environmentally resilient and sustainable crops where they are most needed (Mccouch, 2004; Mccouch et al., 2013). Ultimate progress depends on standardized data collection and reporting, robust cross-environment validation, scalable computing and data infrastructure, and equitable access to AI tools (ownership to openness). Placing greater emphasis on interpretability (XAI) rather than predictive power alone will already enhance biological utility, while collaborative, open, and interoperable datasets and pipelines (Ramírez-Gil et al., 2026) will accelerate model generalization and application—particularly in under-resourced breeding programs.

## Conclusions

The integration of machine learning with high-throughput phenotypic and genotypic data is reshaping plant genetics and crop breeding (Schrider and Kern, 2018). It is increasingly recognized that global food production is both affected by and contributes to long-term environmental shifts (Scherer et al., 2020). These technologies help address the need for varieties capable of withstanding diverse environmental stresses while maintaining high yield. By enabling precise, data-driven selection across a wide range of environments, populations, and omic layers, ML and AI improve the efficiency of identifying superior genotypes. As AI and ML tools become more accessible and training datasets expand, their use in crop improvement programs is expected to grow (Varshney, 2021), supporting sustainable agriculture and global food security (Benitez-Alfonso et al., 2023). Collaboration among breeders, geneticists, eco-physiologist, data scientists, stakeholders, and policymakers will be essential to fully harness these technologies (Mccouch et al., 2020; Branch, 2026). Bridging the gap between genomics (Cañas-Gutierrez et al., 2025), phenomics, computational biology, societal development, and farmer-level technology adoption (Kholova et al., 2024) will also be essential for releasing the full potential of machine learning to enhance plant breeding amid changing environmental conditions and evolving market needs. Because agriculture is both vulnerable to and influential on environmental conditions, integrated ML/AI pipelines combining phenomics, genomics, and multi-omics—paired with practical adoption by breeders and farmers—offer a concrete pathway toward sustainable productivity and resilience. Cross-disciplinary collaboration remains essential for achieving meaningful field-level impact.
